# Lipid profile, lipid ratios, apolipoproteins, and risk of cardiometabolic multimorbidity in men: The Kuopio Ischaemic Heart Disease Risk Factor Study

**DOI:** 10.1002/lipd.12337

**Published:** 2022-01-20

**Authors:** Behnam Tajik, Ari Voutilainen, Jussi Kauhanen, Moshen Mazidi, Gregory Y. H. Lip, Tomi‐Pekka Tuomainen, Masoud Isanejad

**Affiliations:** ^1^ Institute of Public Health and Clinical Nutrition University of Eastern Finland Kuopio Finland; ^2^ Medical Research Council Population Health Research Unit University of Oxford Oxford UK; ^3^ Clinical Trial Service Unit and Epidemiological Studies Unit (CTSU), Nuffield Department of Population Health University of Oxford Oxford UK; ^4^ Department of Twin Research and Genetic Epidemiology King's College London London UK; ^5^ Institute of Life Course and Medical Sciences University of Liverpool Liverpool UK; ^6^ Liverpool Centre for Cardiovascular Sciences University of Liverpool Merseyside Liverpool UK

**Keywords:** apolipoprotein, cardiometabolic multimorbidity, coronary heart disease, diabetes, lipids, stroke

## Abstract

The blood level of lipids, apolipoproteins, and lipid ratios are important predictors of some chronic diseases. However, their association with cardiometabolic multimorbidity (CMM) is less known. We evaluated a wide range of lipid profiles and lipid ratios, including low‐density lipoprotein‐cholesterol (LDL‐C), very‐low‐density lipoprotein‐cholesterol (VLDL‐C), high‐density lipoprotein‐cholesterol (HDL‐C), and apoA1 and B, as well triglyceride and total cholesterol with risk of incident CMM. In 1728 men aged 52.5 ± 5.2 years from the Kuopio Ischaemic Heart Disease were included in this study. We defined CMM as coexisting of two or more of stroke, type 2 diabetes mellitus (T2D), coronary heart disease (CHD). A Cox proportional hazard regression method was applied to evaluate the risk of CMM against the exposures. During the mean follow‐up of 22.4 years, 335 men suffered from CMM conditions. Higher serum triglyceride and VLDL concentrations were associated with a higher risk of coexisting T2D‐CHD (HRs 1.99 (95% CI, 1.12–3.53) and HRs 1.79 (95% CI, 1.04–3.11), respectively. Whereas higher HDL was associated with lower incident [HRs 0.49 (95% CI, 0.40–1.00)]. The HRs for coexisting T2D‐CHD was 2.02 (95% CI, 1.01–3.07) for total cholesterol/HDL‐C, 1.85 (95% CI, 1.04–3.29) for triglyceride/HDL‐C, 1.69 (95% CI, 1.01–2.31) for Non‐HDL‐C/HDL‐C, and 1.89 (95% CI, 1.03–2.46) for apoB/apoA1. In contrast, serum LDL‐C/apoB ratios were inversely associated with the risk of coexisting T2D‐CHD [HRs 0.50 (95% CI, 0.28–0.90)]. No associations were observed between our exposures and other CMM conditions. In conclusion, elevated triglyceride, VLDL‐C, total cholesterol/HDL‐C, TG/HDL‐C, apoB/apoA1 as well as lower LDL‐C/apoB were independently associated with the higher risk of T2D‐CHD coexistence.

**Abbreviations**
Apo‐A1: apolipoprotein A1
ApoB: apolipoprotein B
CHD: coronary heart disease
CMM: cardiometabolic multimorbidity
CVD: cardiovascular disease
HDL: high‐density lipoprotein
KIHD: Kuopio Ischaemic Heart Disease
LDL‐C: low‐density lipoprotein‐cholesterol
VLDL‐C: very‐low‐density lipoprotein‐cholesterol

## INTRODUCTION

Cardiometabolic multimorbidity (CMM) is characterized by the coexistence of two or more diseases including type 2 diabetes (T2D), chronic heart disease (CHD), and stroke, which are among the most prevalent chronic diseases worldwide (Di Angelantonio et al., 2015; Sakakibara et al., 2019). When diseases are co‐occurring and interact with each other, it will significantly aggregate the risk of negative events beyond the sum of the risk of each disease (Di Angelantonio et al., 2015; Rao Kondapally Seshasai et al., 2011). Data from one Finnish study has indicated that T2D patients without previous myocardial infarction have the same risk of mortality from cardiovascular disease (CVD) within 8 years as non‐diabetic patients with prior myocardial infarction (Taskinen, 2002; Taskinen & Borén, 2015).

Dyslipidemia is characterized by the elevation of serum low‐density lipoprotein‐cholesterol (LDL‐C), very low‐density lipoprotein‐cholesterol (VLDL‐C), and lower high‐density lipoprotein‐cholesterol (HDL‐C) (Kopin & Lowenstein, 2017) are important factors in vascular disease as well as with T2D (Taskinen, 2002). Large observational studies have reported a significant association for dyslipidemia with T2D (Mooradian, 2009; Thapa et al., 2017), CHD (Ariyanti & Besral, 2019; Găman et al., 2020), and stroke (Anderson et al., 1987; Kopin & Lowenstein, 2017; Opoku et al., 2019). Non‐HDL cholesterol is also identified as another predictor of CHD risk (Cui et al., 2001). Apolipoprotein A1 (apoA1) and apolipoprotein B (apo B), as two major types of apolipoproteins, are involved in the metabolism of lipids (Casillas‐Muñoz et al., 2018). Abnormal concentrations of apoA1 and apoB are associated with a higher risk of chronic conditions, such as T2D (Siegel et al., 1996), CHD (Sniderman et al., 2019), and stroke (Shilpasree et al., 2013).

Although the importance of dyslipidemia with the single CMM contributing diseases has been studied, the data on the association of lipid, apolipoprotein, and lipid profile ratios when these conditions coexist is less available. This prospective cohort study aimed to evaluate the association of serum lipids and apolipoproteins with the risk of coexisting CMM contributors in middle‐aged adult men belonging to the Kuopio Ischaemic Heart Disease (KIHD) Risk Factor Study.

## METHODS

### Study population

The KIHD study is an ongoing population‐based study that was initially designed to investigate CVD and its risk factors in men from eastern Finland (Salonen, 1988). The baseline examinations were carried out between 1984 and 1989. A total of 2682 men aged 42, 48, 54, or 60 years old at baseline (82.9% of those eligible) participated in this study. The baseline characteristics of the entire study population have been described previously (Salonen et al., 1991). The KIHD protocol was approved by the Research Ethics Committee of the University of Kuopio and complies with the Declaration of Helsinki. All subjects signed written informed consents. Subjects with a history of stroke, diabetes, and CHD (*n* = 855), and missing data on the blood level of lipids, lipoproteins, and apolipoproteins (*n* = 99) were excluded from the analyses, leaving *n* = 1728 men for the analyses.

### Blood lipid measurements

Subjects gave fasting venous blood samples between 8 and 10 A.M. at the baseline examinations. The subjects were instructed to abstain from ingesting alcohol for 3 days and from smoking and eating for 12 h before giving the sample. For blood lipids and lipoprotein fractions (VLDL‐C, LDL‐C, and HDL‐C), fresh serum samples were separated by using ultra‐centrifugation (with a Kontron TGA‐65 ultra‐centrifuge) at 20°C for 10 min as described earlier (Salonen et al., 1991). The VLDL‐C was recovered as the top fraction and HDL was recovered as the supernatant after precipitation of the bottom fraction with dextran sulfate and magnesium chloride. The cholesterol concentration in LDL‐C was calculated as the difference between the bottom and HDL fractions (Salonen et al., 1991). The cholesterol contents of all lipoprotein fractions were measured enzymatically (CHOD‐PAP method, Boehringer Mannheim, Mannheim, FRG) on the day after the last spin. The between‐batch coefficient of variation during 1984–1987 was 2.2% 5.2% for LDL‐C and 9.2% for HDL‐C. For the serum non‐HDL‐C concentration, we used the total cholesterol concentrations minus HDL‐C. ApoA1 and apoB were determined by an immunoturbidimetric method of KONE Oy (Espoo, Finland). In addition, total cholesterol/HDL‐C, triglyceride/HDL‐C, NON‐HDL‐C/HDL‐C, LDL‐C/apoB, apoB/apoA1, HDL‐C/apoA1 ratios were calculated in this study.

### Other measurements

Detailed assessment of the family history of diseases, smoking, alcohol intake, blood pressure, and physical activity is described previously (Laaksonen et al., 2002; Salonen et al., 1992). Serum C‐reactive protein was measured via immunometric assay (IMMULITE High Sensitivity CRP Assay; Diagnostics Products Corporation, USA). BMI was calculated as the ratio of weight (kg) to the square of height in meters. Education was assessed in years by using a self‐administered questionnaire. Hypertension was defined as blood pressure > 140/90 mm Hg or medical treatment for hypertension. Diabetes mellitus was defined as self‐reported diabetes based on previous physician diagnosis or fasting blood glucose of ≥6.7 mmol/L (Salonen et al., 1991). Dietary intakes were assessed with 4‐days food records (Virtanen et al., 2014). Plasma glucose was measured by using a glucose dehydrogenase method after precipitation of proteins by trichloroacetic acid. Serum insulin was determined with a Novo Biolabs radioimmunoassay kit (Novo Nordisk). Insulin resistance and sensitivity and β‐cell function were estimated by the homeostasis model assessment (HOMA) computer algorithm (Wallace & Matthews, 2002). A trained study nurse checked and completed the questionnaires during an interview.

### Ascertainment of follow‐up events

The CMM encompasses metabolic diseases represented by T2D, and CVD represented by CHD and stroke (Di Angelantonio et al., 2015). The follow‐up of those three events was derived from the Finnish inpatient registers of specialized medical care since 1984 together with outpatient registers of specialized medical care since 1998 using the unique personal social security numbers as identifiers (Finnish Institute for Health and Welfare, THL/93.5.05.00/2013). There were no losses to follow‐up, and all diagnoses added from the KIHD baseline until the end of 2018 were included. T2D referred to ICD‐9 codes of 250 and ICD‐10 codes of E11, CHD to ICD‐9 codes of 410–414 and ICD‐10 codes of I20‐25, and stroke to ICD‐9 codes of 430–434 and ICD‐10 codes of I60–64. If a subject had multiple non‐fatal coronary events or non‐fatal strokes during the follow‐up, the first was considered as the endpoint. Regarding CMM cases, we followed the study subject until at least two of the diagnoses, T2D, CHD, and stroke, were assigned, and then we used the date of the second diagnosis as the endpoint date to estimate hazard ratios.

### Statistical analysis

The univariate associations of the serum total cholesterol with demographic, lifestyle, and clinical characteristics at baseline were assessed by means and linear regression for continuous variables and chi‐square test for categorical variables. Cox proportional hazards regression models adjusted for relevant covariates were used to estimate hazard ratios (HRs) of CMM conditions. The validity of the proportional hazard assumption was evaluated by using Schoenfeld residuals, and the assumptions were met. Each lipid and apolipoprotein variable were investigated independently using a separate Cox model adjusted for age and sex. Each continuous variable was scaled to zero mean and unit SD before regression analysis. Thus, the observed hazard ratios denote the relative risk associated with one SD increase in the value of the variable in question. The analyses were controlled for confounders, in two models; model 1 was adjusted for age (years) and examination year. The validity for introducing confounders as continuous variables is tested in the data set (Voutilainen et al., 2021). The multivariable model 2 included model 1 and BMI (kg/m^2^), smoking (pack/years), years of education, leisure‐time physical activity (kilocalories/day), intake of alcohol (grams/week), and use of hypercholesterolemia or hypertension and T2D medications at baseline or during follow‐up (yes or no). Additional adjustments for hypertension, serum blood glucose concentration, hypertension, family history of CHD, T2D or stroke, and income did not appreciably change the associations (<5% change in estimates).

Cohort means were used to replace missing values in covariates (<0.5%). Statistical significance of the interactions on a multiplicative scale was assessed by likelihood ratio tests with a cross‐product term. Tests of linear trend across categories were conducted by assigning the median values for each category of exposure variable and treating those as a single continuous variable. Potential nonlinear associations were assessed semi‐parametrically by using restricted cubic splines.

To control the probability of committing any type I error with multiple testing, we used Bonferroni correction (Schober & Vetter, 2020). The significance threshold was set as *α* less than 0.020, derived by dividing *p*‐value for the number of hypothesis tests. Only test results with *p*‐values smaller than the adjusted significance criterion are considered statistically significant. Data were analyzed using the SPSS software version 27 for Windows (Armonk, NY: IBM Corp.).

## RESULTS

### Baseline characteristic

During the mean follow‐up of 22.4 years of 1728 men, the highest frequency of coexisting conditions was for the stroke‐CHD group (*n* = 146) as compared to T2D‐CHD (*n* = 110), T2D‐stroke (*n* = 50), and T2D‐CHD‐stroke groups (*n* = 29). Baseline characteristics of the participants according to the CMM conditions are presented in Table 1. Men with T2D‐CHD coexistence had higher BMI, alcohol intake, less physically active, and had lower serum concentrations of TG, total cholesterol, VLDL‐C, and LDL, as compared to other CMM conditions; however, men with all three conditions were less educated, and have higher systolic blood pressure (Table 1).

**TABLE 1:** Baseline characteristics according to cardiometabolic multimorbidity subgroups

	Cardiometabolic multimorbidity subgroups			
Variables	CHD + stroke (*n* = 146)	CHD + T2D (*n* = 110)	Stroke + T2D (*n* = 50)	All three (*n* = 29)
Age (years)	53.2 (4.1)	52.8 (4.5)	53.4 (3.6)	53.9 (3.87)
Education (years)	9.5 (3.7)	9.0 (3.8)	8.7 (3.4)	8.0 (2.9)
BMI (kg/m^2^)	27.6 (3.4)	28.6 (3.5)	27.9 (2.8)	28.2 (3.0)
Smoking (pack/years)	30.5%	30.1%	34.7%	35.9%
Leisure‐time physical activity (kcal/days)	135.5 (167.0)	122.7 (170.9)	126.9 (210.5)	139.4 (265.1)
Serum triglyceride (mmol/L)	1.29 (0.83)	1.67 (1.32)	1.41 (0.78)	1.45 (0.85)
Serum total cholesterol (mmol/L)	5.85 (1.02)	6.00 (1.11)	5.85 (0.98)	5.66 (1.00)
Serum VLDL‐C (mmol/L)	0.55 (0.39)	0.71 (0.65)	0.61 (0.37)	0.62 (0.37)
Serum LDL cholesterol (mmol/L)	4.01 (0.98)	4.06 (1.03)	3.99 (0.94)	3.79 (0.92)
Serum HDL cholesterol (mmol/L)	1.28 (0.31)	1.22 (0.29)	1.23 (0.26)	1.20 (0.28)
Serum non‐HDL‐C (mmol/L)	4.57 (0.50)	4.78 (0.63)	4.68 (0.58)	4.41 (0.49)
Serum apoA1 (g/L)	1.35 (0.26)	1.30 (0.29)	1.30 (0.24)	1.28 (0.27)
Serum apoB (g/L)	1.00 (0.22)	1.07 (0.23)	1.03 (0.23)	0.98 (0.22)
C‐reactive protein (mmol/L)	2.49 (4.22)	2.28 (3.24)	2.09 (4.07)	2.44 (5.27)
Systolic blood pressure (mm Hg)	137 (12)	136 (16)	136 (12)	139 (16)
Diastolic blood pressure (mm Hg)	92 (10)	92 (9)	92 (9)	94 (8)
Alcohol intake (g/days)	65.2 (91.7)	72.0 (105.7)	43.6 (61.3)	45.5 (61.3)
Medication^a^ (%)	80.1%	83.4%	86.1%	84.1%

Abbreviations: Apo‐A, apolipoprotein A1; ApoB, apolipoprotein B; BMI, body mass index; CHD, coronary heart disease; HDL‐C, high‐density lipoprotein‐cholesterol; LDL‐C, low‐density lipoprotein‐cholesterol; VLDL‐C, very‐low‐density lipoprotein‐cholesterol; T2D, type 2 diabetes mellitus.

*Note*: Results are means (SD) for continuous variables and percentages for categorical data.

^a^ Medication includes antihypertensive, anti‐hypercholesterolemia, and type II diabetes medications.

### Serum triglyceride

Serum triglyceride concentrations were associated with a higher risk of CMM, for those with coexisting T2D‐CHD [(extreme‐quartile multivariate‐adjusted HRs 1.99 (95% CI, 1.12–3.53, *p* for trend = 0.001) (Model 2, Table 2). No statistically significant associations were found between serum triglyceride concentration and other CMM conditions (Table 2). Further analysis using the continuous value of triglyceride showed similar results.

**TABLE 2:** Extreme quartile hazards ratios for cardiometabolic multimorbidity according to serum lipids, lipoproteins, and apolipoproteins concentrations

	Cardiometabolic multimorbidity subgroups			
	CHD + stroke	CHD + T2D	Stroke + T2D	CHD + stroke + T2D
Triglyceride (mmol/L)	1.29 (0.83)^a^	1.67 (1.32)	1.41 (0.78)	1.45 (0.85)
Model 1^b^	1.22 (0.75–2.00)^c^	2.95 (1.69–5.14)*	2.23 (0.83–5.19)	1.56 (0.53–4.59)
Model 2^d^	0.98 (0.59–1.63)	1.99 (1.12–3.53)*	1.66 (0.68–4.04)	1.18 (0.39–3.57)
Total cholesterol (mmol/L)	5.86 (1.44)	6.04 (1.53)	5.86 (1.45)	5.76 (1.38)
Model 1	1.01 (0.62–1.64)	1.40 (0.82–2.39)	0.88 (0.36–2.12)	0.58 (0.17–1.99)
Model 2	0.96 (0.59–1.56)	1.35 (0.79–2.31)	0.86 (0.36–2.08)	0.57 (0.17–1.98)
VLDL‐C (mmol/L)	0.55 (0.39)	0.71 (0.65)	0.61 (0.37)	0.62 (0.37)
Model 1	1.25 (0.78–2.00)	2.31 (1.36–3.95)*	2.01 (0.93–4.36)	2.27 (0.84–6.14)
Model 2	1.11 (0.69–1.79)	1.79 (1.04–3.11)*	1.65 (0.75–3.65)	1.93 (0.66–5.06)
LDL‐C (mmol/L)	4.01 (0.98)	4.06 (1.03)	3.99 (0.94)	3.79 (0.92)
Model 1	1.07 (0.67–1.70)	1.10 (0.65–1.87)	1.10 (0.50–2.43)	1.07 (0.15–1.56)
Model 2	0.98 (0.62–1.57)	1.01 (0.59–1.72)	1.04 (0.47–2.31)	1.15 (0.14–1.51)
HDL‐C (mmol/L)	1.28 (0.31)	1.23 (0.29)	1.23 (0.26)	1.20 (0.29)
Model 1	0.85 (0.53–1.35)	0.47 (0.28–0.80)*	0.47 (0.21–1.06)	0.39 (0.14–1.10)
Model 2	1.03 (0.64–1.66)	0.49 (0.40–1.00)*	0.68 (0.30–1.54)	0.53 (0.18–1.52)
Non‐HDL‐C (mmol/L)	4.57 (0.91)	4.78 (1.13)	4.62 (1.02)	4.45 (1.03)
Model 1	1.06 (0.65–1.74)	1.44 (0.87–2.41)	0.81 (0.35–1.83)	0.49 (0.15–1.65)
Model 2	0.96 (0.59–1.57)	1.25 (0.75–2.08)	0.69 (0.30–1.58)	0.45 (0.14–1.53)
ApoA1 (g/L)	1.35 (0.26)	1.30 (0.29)	1.30 (0.24)	1.28 (0.26)
Model 1	0.86 (0.52–1.42)	0.44 (0.25–0.77)*	0.78 (0.35–1.76)	0.44 (0.15–1.33)
Model 2	1.01 (0.61–1.68)	0.61 (0.35–1.07)	1.11 (0.48–2.54)	0.59 (0.19–1.77)
ApoB (g/L)	1.00 (0.22)	1.07 (0.23)	1.03 (0.23)	0.98 (0.22)
Model 1	0.98 (0.60–1.59)	1.71 (1.00–2.92)	1.17 (0.53–2.57)	0.70 (0.25–1.98)
Model 2	0.68 (0.30–1.58)	1.35 (0.78–2.32)	1.01 (0.46–2.22)	0.62 (0.22–1.76)

Abbreviations: Apo‐A, apolipoprotein A1; ApoB, apolipoprotein B; BMI, body mass index; CHD, coronary heart disease; HDL‐C, high‐density lipoprotein‐cholesterol; LDL‐C, low‐density lipoprotein‐cholesterol; VLDL‐C, very‐low‐density lipoprotein‐cholesterol; T2D, type 2 diabetes mellitus.

^a^ Mean (SD).

^b^ Model 1: adjusted for age and examination year.

^c^ Values are hazard ratios (95% confidence interval).

^d^ Model 2: adjusted for model 1 plus body mass index, smoking, leisure‐time physical activity, education, income, medication, alcohol intake and energy intake.

* *p*‐value ≤ 0.020.

### Total cholesterol, VLDL‐C, LDL‐C, HDL‐C, and non‐HDL‐C

Serum VLDL‐C concentration was directly associated with the risk of CMM only for those with coexisting T2D‐CHD [extreme‐quartile multivariate‐adjusted HRs 1.79 (95% CI, 1.04–3.11, *p* for trend = 0.008] (Model 2, Table 2). In contrast, higher serum HDL‐C concentration was associated with 42% lower HR of coexisting T2D‐CHD [extreme‐quartile multivariate‐adjusted HRs were 0.49 (95% CI, 0.40–1.00, *p* for trend 0.04)] (Model 2, Table 2). No associations were observed between the serum HDL‐C concentration and other CMM conditions (Table 2). There was no statistically significant association in the analysis between serum total cholesterol, LDL‐C, and non‐HDL‐C concentrations and CMM conditions (Table 2). When evaluating the variables continuously, similar associations were observed between serum total cholesterol, VLDL‐C, LDL‐C, HDL‐C, non‐HDL‐C concentrations, and likelihood of CMM.

### ApoA1 and ApoB

After adjustments for the age and examination (Model 1), In the highest versus the lowest serum apoA1 concentration, the CMM (T2D‐CHD) HR was decreased by 66% [HR was 0.44 (95% CI, 0.25–0.77, *p* for trend 0.006)]; however, further adjustment attenuated the association [multivariate‐adjusted HR was 0.61 (95% CI, 0.35–1.07, *p* for trend 0.09)]. No statistically significant associations were observed between serum apoB concentration and coexisting CMM conditions when introducing categorical or continuous variables.

### Lipid ratios

The association between lipid ratios with coexisting CMM conditions was evaluated by comparing the lowest versus highest quartiles. Higher serum LDL‐C/apoB ratios were associated with a lower HR of coexisting T2D‐CHD [extreme‐quartile multivariate‐adjusted HRs 0.50 (95% CI, 0.28–0.90 *p* for trend = 0.030] (Model 2, Table 3). The HRs for coexisting T2D‐CHD in the multivariate‐adjusted model (Model 2) was 1.69 (95% CI, 1.05–2.89, *p* for trend 0.05) for cholesterol/HDL‐C, 1.85 (95% CI, 1.04–3.29, *p* for trend 0.002) for triglyceride/HDL‐C, 1.69 (95% CI, 1.01–2.31, *p* for trend 0.03) for non‐HDL‐C/HDL‐C, and 1.89 (95% CI, 1.03–2.46, *p* for trend 0.05) for apoB/apoA1 (Model 2, Table 3). No statistically significant associations were found between serum lipid ratios and other CMM conditions (Table 3). Similar associations were observed when we evaluated them continuously.

**TABLE 3:** Extreme quartile hazards ratios for cardiometabolic multimorbidity according to serum lipids, lipoproteins, and apolipoproteins ratios

	Cardiometabolic multimorbidity subgroups			
	CHD + stroke	CHD + T2D	Stroke + T2D	CHD + stroke + T2D
LDL‐C/ApoB	4.02 (0.54)^a^	3.80 (0.53)	3.88 (0.52)	3.89 (0.50)
Model 1^b^	1.30 (0.80–2.11)^c^	0.39 (0.22–0.68)*	0.60 (0.27–1.36)	0.71 (0.24–2.15)
Model 2^d^	1.50 (0.90–2.50)	0.50 (0.28–0.90)*	0.71 (0.31–1.63)	0.85 (0.28–2.63)
Total cholesterol/HDL‐C	4.82 (1.44)	5.14 (1.53)	4.98 (1.45)	4.94 (1.48)
Model 1	1.07 (0.69–1.68)	2.48 (1.38–4.46)*	1.26 (0.54–2.91)	1.45 (0.46–4.59)
Model 2	0.85 (0.54–1.33)	2.02 (1.01–3.07)*	0.86 (0.37–2.03)	1.08 (0.34–3.45)
Triglyceride/HDL‐C	1.11 (0.83)	1.54 (1.49)	1.28 (0.98)	1.37 (1.08)
Model 1	1.32 (0.83–2.10)	2.91 (1.67–5.06)*	2.17 (0.87–5.45)	2.29 (0.77–6.79)
Model 2	1.02 (0.63–1.65)	1.85 (1.04–3.29)*	1.48 (0.58–3.83)	1.59 (0.52–4.90)
Non‐HDL‐C/HDL‐C	3.82 (1.34)	4.14 (1.53)	3.98 (1.45)	3.94 (1.48)
Model 1	1.07 (0.69–1.68)	2.48 (1.38–4.46)*	1.26 (0.54–2.91)	1.45 (0.46–4.59)
Model 2	0.85 (0.54–1.33)	1.69 (1.01–2.31)*	0.86 (0.37–2.03)	1.08 (0.34–3.45)
VLDL‐C/TG	0.43 (0.19)	0.42 (0.19)	0.42 (0.18)	0.44 (0.16)
Model 1	0.93 (0.57–1.52)	0.73 (0.41–1.28)	0.81 (0.32–2.07)	1.05 (0.30–3.64)
Model 2	0.97 (0.60–1.61)	0.73 (0.43–1.35)	0.82 (0.32–2.09)	1.04 (0.30–3.63)
ApoB/ApoA1	0.77 (0.21)	0.85 (0.22)	0.81 (0.21)	0.79 (0.22)
Model 1	1.01 (0.64–1.60)	2.69 (1.48–4.87)*	2.18 (0.91–5.22)	1.42 (0.51–4.00)
Model 2	0.81 (0.51–1.30)	1.89 (1.03–2.46)*	1.56 (0.64–2.79)	1.05 (0.37–2.04)
HDL‐C/ApoA1	0.95 (0.15)	0.95 (0.16)	0.95 (0.17)	0.95 (0.15)
Model 1	0.85 (0.52–1.40)	0.54 (0.30–0.97)*	0.57 (0.23–1.41)	0.63 (0.20–2.12)
Model 2	0.99 (0.60–1.67)	0.71 (0.40–1.27)	0.75 (0.29–1.80)	0.79 (0.23–2.78)

Abbreviations: Apo‐A, apolipoprotein A1; ApoB, apolipoprotein B; BMI, body mass index; CHD, coronary heart disease; HDL‐C, high‐density lipoprotein‐cholesterol; LDL‐C, low‐density lipoprotein‐cholesterol; VLDL‐C, very‐low‐density lipoprotein‐cholesterol; T2D, type 2 diabetes mellitus.

^a^ Mean (SD).

^b^ Model 1: adjusted for age and examination year.

^c^ Values are hazard ratios (95% confidence interval).

^d^ Model 2: adjusted for model 1 plus body mass index, smoking, leisure‐time physical activity, education, income, medication, alcohol intake, and energy intake.

* *p*‐value ≤ 0.020.

## DISCUSSION

In this prospective study of 1728 middle‐aged and older men, the primary finding indicated that higher quartiles of serum triglyceride and VLDL‐C concentrations were significantly associated with the risk of CMM (T2D‐CHD) coexistence, whereas HDL‐C reduced the risk. Higher serum LDL‐C/apoB ratios were associated with a lower risk of CMM (coexisting T2D‐CHD). Direct associations between higher cholesterol/HDL‐C, triglyceride/HDL‐C, Non‐HDL‐C/HDL‐C, and apoB/apo‐A1 ratios with CMM (T2D‐CHD coexistence) were observed. Data from previous studies were mostly available for single/individual diseases, while our prospective study provides new evidence that risk of evaluating CM outcomes in combination is clinically relevant.

Systemic reviews have shown moderate and highly significant associations between triglyceride values and CHD risk (Sarwar et al., 2007), and a higher triglyceride level with increased risk of CVD mainly due to an increased risk of CHD but not a stroke (Ye et al., 2019), where triglyceride elevated levels were not associated with the risk of CVD among T2D patients. After adjusting for coronary risk factors, especially HDL‐C cholesterol, in this study elevated triglyceride levels were significantly associated with a higher risk of CMM (T2D‐CHD coexistence). Diabetes‐associated atherosclerosis factors, such as hypertriglyceridemia, can cause insulin resistance, which impairs glucose metabolism and elevate the accumulation of fatty acid stratification metabolites (Lee et al., 2018; Robertson et al., 2004). Altogether, these impairments are significant risk factors for CVD. In addition, insulin resistance in T2D patients increases both inflammation and atherosclerosis (Kahn et al., 2000; Trovati et al., 1988; Defronzo, 2009). Moreover, elevated triglyceride influences the production of proinflammatory cytokines, fibrinogen and coagulation factors, and impairment of fibrinolysis, hence, increasing the process of atherogenesis (Kim et al., 2017; Zheng et al., 2018).

Overall, data regarding triglyceride with CMM and as a CHD risk factor among T2D patients are scarce. A cross‐sectional study showed higher triglyceride levels were associated with an increased risk of CVD among patients with a short duration of diabetes, whereas lower triglyceride levels reduced the risk of CVD with a longer duration of diabetes (Ren et al., 2018). Similarly, our study population were relatively younger and living with T2D for a shorter time. It may be that elevated triglyceride levels increased the risk of CVD mainly due to association with CHD among T2D patients.

Considering that TGs in plasma are transported by a mixture of different lipoprotein species, such as CMs, VLDLs, and VLDL remnants, we have identified some of the selective measurements of these TG‐rich lipoproteins to obtain further insight into TG association with CMM. The present study showed no significant association for VLDL/TG, while a previous large study found that VLDL‐C/TG was linked with coronary artery disease (Hopkins et al., 2009). This difference might be because we investigated CM coexisting risk in relatively healthy adults, and Hopkins et al. had a large sample with elevated coronary artery disease risk. Furthermore, triglyceride/HDL‐C (Yang et al., 2019) ratios have been associated with insulin resistance (Gonzalez et al., 2008; Quispe et al., 2015); obesity (Karelis et al., 2007), metabolic syndrome, and cardiovascular events, and it has been suggested to add values by reflecting the complex interactions of lipoprotein metabolism and predict plasma atherogenicity (Millán et al., 2009). In our study, higher triglyceride/HDL‐C ratios were associated with the T2D‐CHD coexistence. The enrichment of HDL‐C by the high content of triglyceride leads to the formation of more numerous and denser LDL particles. Our finding agrees with studies showing that the triglyceride/HDL‐C ratio can act as a predictor for CHD (Hadaegh et al., 2009) and T2D (Young et al., 2019).

This study also showed a higher risk of CMM (T2D‐CHD coexistence) in those with a higher quartiles of VLDL‐C but not with LDL‐C. These findings concord with the mechanism by which triglyceride involves in altering LDL‐particle size. The LDL‐particle size has been associated negatively with serum triglyceride levels (Gong et al., 2016), meaning that when the triglyceride levels are elevated, the LDL‐particle sizes became smaller. Of note, the Québec Cardiovascular Study demonstrated that patients had LDL‐particle size of 25.5 nm or smaller, the CHD incidence increased significantly as the serum LDL‐C level increases, while in patients having large LDL‐particle sizes of 26.0 nm or greater, no significant difference in coronary artery disease events was observed according to the absolute serum LDL‐C level (St‐Pierre et al., 2005). VLDL‐C is a triglyceride‐rich particle and shown to be associated with the progression of atherosclerosis (Lee et al., 2017).

Another important alternative index for LDL‐particle size is the ratio to apoB (i.e., LDL‐C/apoB) (Millán et al., 2009), and the best tool to identify patients with small dense LDL particles (phenotype B) (Wägner et al., 2002). Our results indicate a significant inverse relationship between the LDL‐C/apoB ratio and the risk of T2D‐CHD coexistence, demonstrate that the risk of CMM increases as LDL size decreases.

Our finding also suggested that higher serum HDL‐C levels may lower the risk of T2D‐CHD coexistence. By and large, there is a gender difference in HDL‐C, the levels are higher in women than in men (Expert Panel on Detection, 2001, Li et al., 2016), which should be considered when interpreting the results. Non‐HDL cholesterol (i.e., total cholesterol − HDL‐C) has been proposed for the identification of dyslipidemia as an alternative to the classic lipid risk factors, as well as HDL‐C (Expert Panel on Detection, 2001, Di Bonito et al., 2015). In the KIHD data, there was no significant association between non‐HDL cholesterol and CMM conditions. Smaller cholesterol‐poor HDL particles are reflected by lower HDL‐C levels and HDL‐C/apoA‐1 ratios. However, our study showed no significant association for HDL‐C/apoA1 with CMM conditions.

In addition, analyses of the KIHD data showed that the apoB/apoA1 ratio was associated with CMM (T2D‐CHD coexistence). Findings from large studies have shown an association between the apoB/apo‐A1 ratio and the prediction of cardiovascular risk beyond TG and cholesterol (Casillas‐Muñoz et al., 2018). In this study, the apoB/apo‐A1 ratio was also associated with single outcomes of T2D‐CHD. ApoB and apoA1 are transporting components of atherogenic (LDL‐C, VLDC‐C, and IDL) and antiatherogenic (HDL‐C) lipid profiles, respectively (Casillas‐Muñoz et al., 2018).

Our investigation for apoA1 and apoB showed no statistically significant association with the coexisting conditions, but several studies included in meta‐analysis demonstrate the links between apoB and CHD, showing the ability of apoB to predict CHD (Parish et al., 2009), which was also detected in our study (results not shown).

In the present study, significant associations were observed only for the coexisting T2D‐CHD, and not for other conditions. This might be because the relatively lower number of the participants in other CMM groups. Our analysis did not show statistically significant differences between these groups, but there might be other factors that we could not control in this study. It might also be due to artery lipid accumulation and insulin resistance complications in patients with T2D that are also underlying conditions for cardiovascular diseases (Laakso & Kuusisto, 2014), which are more apparent in these patients. For example, TG/HDL in this study is a variable representing diabetic dyslipidemia and a marker for insulin resistance (Scicali et al., 2021), it may be that in patients with coexisting T2D and CHD, such blood biomarker was more augmented. However, further studies are warranted to consider CM outcomes in co‐existence as it is present in patients in the clinical settings.

We acknowledge that our study has limitations. Hypertension has a central role in cardiometabolic disease and stroke and it is usually associated with metabolic disorders, such as insulin resistance, obesity, and dyslipidemia (Tasic & Lovic, 2018). We defined CMM without hypertension history as a disease because when using elevated blood pressure as a binary variable can reduce the effect of blood pressure on chronic disease (Di Angelantonio et al., 2015; Lewington et al., 2002). Given the younger mean age of our study population, the prevalence of the coexisting conditions may be expected to be lower. This study was only included men and given the discrepancy existing for dyslipidemia among the men and women, consideration should be taken while interpreting our results. Also, because of the observational study design, conclusions about causality cannot be drawn. Noteworthy that the definition of CMM are varied in previous studies, we have developed our method for cardiometabolic diseases ascertainment according to prior study (Di Angelantonio et al., 2015).

Elevated triglyceride, VLDL‐C, total cholesterol/HDL‐C, TG/HDL‐C, apoB/apoA1 as well as lower LDL‐C/apoB were independently associated with the higher risk of T2D‐CHD coexistence among middle‐aged and older men. These results upgrade the importance of triglyceride and VLDL‐C, and most importantly the LDL particle size, even in the younger age, and to utilize these measures as independent predictors of CMM, especially T2D‐CHD coexistence.

## CONFLICT OF INTEREST

The authors declare that they have no conflict of interest.

## ETHICS STATEMENT

The authors declare that they have no conflict of interest and fulfill all ethical statement rules. The KIHD protocol was approved by the Research Ethics Committee of the University of Kuopio and complies with the Declaration of Helsinki.

## AUTHOR CONTRIBUTIONS

Behnam Tajik and Tomi‐Pekka Tuomainen acquired the data and designed and conducted the research; Behnam Tajik and Masoud Isanejad analyzed the data and drafted the manuscript. Ari Voutilainen was consulted for the data analysis approach. Tomi‐Pekka Tuomainen, Ari Voutilainen, Moshen Mazidi, Gregory Y. H. Lip critically revised the manuscript for important intellectual content. All authors have read and provided an intellectual review of this study.
